# 2-[(*E*)-2-(4-Ethoxy­phen­yl)ethen­yl]-1-methyl­pyridinium 4-bromo­benzene­sulfonate monohydrate^1^

**DOI:** 10.1107/S1600536809055846

**Published:** 2010-01-09

**Authors:** Hoong-Kun Fun, Kullapa Chanawanno, Suchada Chantrapromma

**Affiliations:** a X-ray Crystallography Unit, School of Physics, Universiti Sains Malaysia, 11800 USM, Penang, Malaysia; b Crystal Materials Research Unit, Department of Chemistry, Faculty of Science, Prince of Songkla University, Hat-Yai, Songkhla 90112, Thailand

## Abstract

In the title compound, C_16_H_18_NO^+^·C_6_H_4_BrO_3_S^−^·H_2_O, the cation exists in an *E* configuration with respect to the ethenyl bond and is slightly twisted with a dihedral angle of 8.5 (2)° between pyridinium and benzene rings. In the crystal, the cations are arranged in layers parallel to (100), with π–π inter­actions between pyridinium and benzene rings [centroid–centroid distances = 3.651 (3) and 3.613 (3) Å]. The anions and water mol­ecules are located between the cationic layers. The ions and water mol­ecules are linked into a three-dimensional framework by O—H⋯O and C—H⋯O hydrogen bonds.

## Related literature

The title compound was synthesized as part of an investigation of the influence of the counter-ions on non-linear optical (NLO) properties. For background to NLO materials research, see: Coe *et al.* (2002 ▶); Pan *et al.* (1996 ▶). For related structures, see: Chanawanno *et al.* (2009 ▶); Chantrapromma *et al.* (2006 ▶, 2009 ▶); Laksana *et al.* (2008 ▶). For bond-length data, see: Allen *et al.* (1987 ▶). For the stability of the temperature controller used in the data collection, see: Cosier & Glazer (1986 ▶).
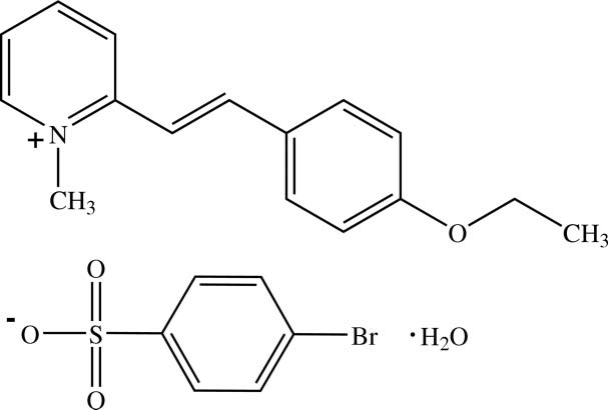


## Experimental

### 

#### Crystal data

C_16_H_18_NO^+^·C_6_H_4_BrO_3_S^−^·H_2_O
*M* *_r_* = 494.39
Monoclinic, 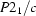

*a* = 9.8022 (5) Å
*b* = 6.5162 (3) Å
*c* = 34.9982 (17) Å
β = 105.102 (3)°
*V* = 2158.24 (18) Å^3^
*Z* = 4
Mo *K*α radiation
μ = 2.04 mm^−1^
*T* = 100 K
0.34 × 0.31 × 0.19 mm

#### Data collection

Bruker APEXII CCD area-detector diffractometer
Absorption correction: multi-scan (*SADABS*; Bruker, 2005 ▶) *T*_min_ = 0.547, *T*_max_ = 0.703
30564 measured reflections
6286 independent reflections
4937 reflections with *I* > 2σ(*I*)
*R*_int_ = 0.076

#### Refinement

*R*[*F* ^2^ > 2σ(*F* ^2^)] = 0.071
*wR*(*F* ^2^) = 0.224
*S* = 1.15
6286 reflections
275 parameters
H-atom parameters constrained
Δρ_max_ = 1.26 e Å^−3^
Δρ_min_ = −1.36 e Å^−3^

### 

Data collection: *APEX2* (Bruker, 2005 ▶); cell refinement: *SAINT* (Bruker, 2005 ▶); data reduction: *SAINT*; program(s) used to refine structure: *SHELXTL* (Sheldrick, 2008 ▶); molecular graphics: *SHELXTL*; software used to prepare material for publication: *SHELXTL* and *PLATON* (Spek, 2009 ▶).

## Supplementary Material

Crystal structure: contains datablocks global, I. DOI: 10.1107/S1600536809055846/ci5012sup1.cif

Structure factors: contains datablocks I. DOI: 10.1107/S1600536809055846/ci5012Isup2.hkl

Additional supplementary materials:  crystallographic information; 3D view; checkCIF report

## Figures and Tables

**Table 1:** Hydrogen-bond geometry (Å, °)

*D*—H⋯*A*	*D*—H	H⋯*A*	*D*⋯*A*	*D*—H⋯*A*
O1*W*—H2*W*1⋯O4	0.85	2.09	2.929 (6)	171
O1*W*—H1*W*1⋯O2^i^	0.85	1.99	2.827 (6)	168
C1—H1*A*⋯O1*W*^ii^	0.93	2.23	3.154 (7)	176
C2—H2*A*⋯O1*W*^iii^	0.93	2.43	3.223 (7)	143
C4—H4*A*⋯O4	0.93	2.50	3.378 (7)	158
C6—H6*A*⋯O3^iv^	0.93	2.56	3.442 (7)	159
C13—H13*A*⋯O3^iv^	0.93	2.49	3.387 (7)	161
C14—H14*A*⋯O2^v^	0.96	2.57	3.384 (7)	143
C14—H14*C*⋯O3^iv^	0.96	2.51	3.129 (7)	122

Symmetry codes: (i) 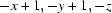; (ii) 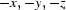; (iii) 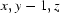; (iv) 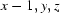; (v) 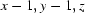.

**Fig. 1.**
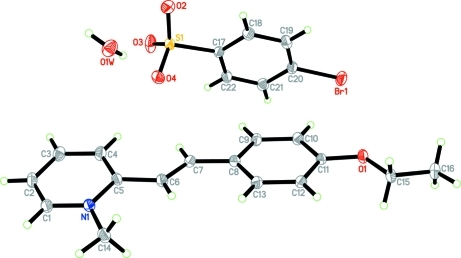
The asymmetric unit of the title compound, with 50% probability displacement ellipsoids and the atom-numbering scheme.

**Fig. 2.**
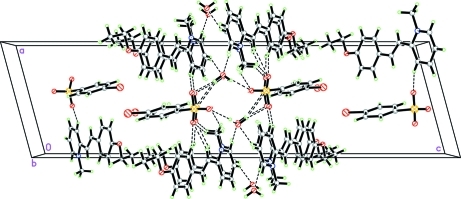
The crystal packing of the title compound, viewed down the b axis. Hydrogen bonds are shown as dashed lines.

## supplementary crystallographic information

### Comment

Ionic organic crystals are of special interest due to their high second order optical nonlinearities (Coe *et al.*, 2002). The orientation of ionic chromophores can be arranged simply by changing the counter-ions (Pan *et al.*, 1996). During the course of our NLO materials research, we have previously synthesized and reported crystal structures of related pyridinium salts containing the 2-[(*E*)-2-(4-ethoxyphenyl)ethenyl]-1-methylpyridinium cationic part (Chanawanno *et al.*, 2009; Laksana *et al.*, 2008). The title compound was synthesized by retaining the same cationic part but changing the anion counter part to 4-bromobenzenesulfonate in order to investigate the influence of the counter-ions on the NLO properties. However, it was found that the title compound crystallized in a centrosymmetric space group *P*2_1_/*c* and hence no second-order nonlinear optical properties are observed.

In the title compound (Fig. 1), the cation exists in an *E* configuration with respect to the ethenyl bond [C5—C6—C7—C8 = -179.9 (5)°]. The cation is slightly twisted with a dihedral angle between the pyridinium and benzene rings of 8.5 (2)°. The pyridinium and benzene rings of the cation form dihedral angles of 79.2 (2) and 71.0 (2)°, respectively, with the benzene ring of the anion. Bond distances in both cation and anion have normal values (Allen *et al.*, 1987) and are comparable to those observed in related structures (Chanawanno *et al.*, 2009; Chantrapromma *et al*., 2009; Laksana *et al.*, 2008).

In the crystal, the cations are stacked along the *b* axis and are arranged in layers parallel to the (100) with π–π interactions involving pyridinium (centroid Cg1) and benzene (centroid Cg2) rings [Cg1···Cg1^ii^ = 3.651 (3) Å and Cg1···Cg2^iii^ = 3.613 (3) Å; symmetry codes as in Table 1]. The anions and water molecules are located between the cationic layers. The cations are linked with the water molecules and anions by C—H···O weak interactions (Table 1), whereas the anions are linked with water molecules by O—H···O hydrogen bonds (Table 1). These interactions connect the ionic units and water molecules into a three-dimensional network (Fig. 2).

### Experimental

2-[(*E*)-2-(4-Ethoxyphenyl)ethenyl]-1-methylpyridinium iodide (0.21 g, 0.58 mmol) which was prepared according to the previous method (Laksana *et al.*, 2008) was mixed with silver 4-bromobenzenesulfonate (Chantrapromma *et al.*, 2006) (0.20 g, 0.58 mmol) in methanol (100 ml) and stirred for 0.5 h. The precipitate of silver iodide which formed was filtered and the filtrate was evaporated to give the title compound as a yellow solid. Yellow block-shaped single crystals of the title compound suitable for *X*-ray structure determination were recrystallized from methanol by slow evaporation at room temperature over a few weeks (m.p. 463-465 K).

### Refinement

H atoms were positioned geometrically and allowed to ride on their parent atoms, with O–H = 0.85 Å and C–H = 0.93-0.97 Å. The *U*_iso_ values were constrained to be 1.5*U*_eq_ of the carrier atom for methyl H atoms and 1.2*U*_eq_ for the remaining H atoms. A rotating group model was used for the methyl groups. The highest residual electron density peak is located at 0.81 Å from Br1 and the deepest hole is located at 1.90 Å from Br1.

### Crystal data

**Table:**

C_16_H_18_NO^+^·C_6_H_4_BrO_3_S^−^·H_2_O	*F*(000) = 1016
*M**_r_* = 494.39	*D*_x_ = 1.522 Mg m^−^^3^
Monoclinic, *P*2_1_/*c*	Melting point = 463–465 K
Hall symbol: -P 2ybc	Mo *K*α radiation, λ = 0.71073 Å
*a* = 9.8022 (5) Å	Cell parameters from 6286 reflections
*b* = 6.5162 (3) Å	θ = 2.4–30.0°
*c* = 34.9982 (17) Å	µ = 2.04 mm^−^^1^
β = 105.102 (3)°	*T* = 100 K
*V* = 2158.24 (18) Å^3^	Block, yellow
*Z* = 4	0.34 × 0.31 × 0.19 mm

### Data collection

**Table:**

Bruker APEXII CCD area-detector diffractometer	6286 independent reflections
Radiation source: sealed tube	4937 reflections with *I* > 2σ(*I*)
graphite	*R*_int_ = 0.076
φ and ω scans	θ_max_ = 30.0°, θ_min_ = 2.4°
Absorption correction: multi-scan (*SADABS*; Bruker, 2005)	*h* = −13→12
*T*_min_ = 0.547, *T*_max_ = 0.703	*k* = −7→9
30564 measured reflections	*l* = −49→49

### Refinement

**Table:**

Refinement on *F*^2^	Primary atom site location: structure-invariant direct methods
Least-squares matrix: full	Secondary atom site location: difference Fourier map
*R*[*F*^2^ > 2σ(*F*^2^)] = 0.071	Hydrogen site location: inferred from neighbouring sites
*wR*(*F*^2^) = 0.224	H-atom parameters constrained
*S* = 1.15	*w* = 1/[σ^2^(*F*_o_^2^) + (0.0686*P*)^2^ + 18.6991*P*] where *P* = (*F*_o_^2^ + 2*F*_c_^2^)/3
6286 reflections	(Δ/σ)_max_ = 0.001
275 parameters	Δρ_max_ = 1.25 e Å^−^^3^
0 restraints	Δρ_min_ = −1.36 e Å^−^^3^

### Special details

**Table:**

Experimental. The crystal was placed in the cold stream of an Oxford Cryosystems Cobra open-flow nitrogen cryostat (Cosier & Glazer, 1986) operating at 100.0 (1) K.
Geometry. All esds (except the esd in the dihedral angle between two l.s. planes) are estimated using the full covariance matrix. The cell esds are taken into account individually in the estimation of esds in distances, angles and torsion angles; correlations between esds in cell parameters are only used when they are defined by crystal symmetry. An approximate (isotropic) treatment of cell esds is used for estimating esds involving l.s. planes.
Refinement. Refinement of F^2^ against ALL reflections. The weighted R-factor wR and goodness of fit S are based on F^2^, conventional R-factors R are based on F, with F set to zero for negative F^2^. The threshold expression of F^2^ > 2sigma(F^2^) is used only for calculating R-factors(gt) etc. and is not relevant to the choice of reflections for refinement. R-factors based on F^2^ are statistically about twice as large as those based on F, and R- factors based on ALL data will be even larger.

### Fractional atomic coordinates and isotropic or equivalent isotropic displacement parameters (Å^2^)

**Table:**

	*x*	*y*	*z*	*U*_iso_*/*U*_eq_	
Br1	0.61259 (6)	1.18042 (10)	0.235458 (16)	0.02660 (17)	
S1	0.57829 (13)	0.5608 (2)	0.09005 (4)	0.0181 (3)	
O1	0.0959 (4)	1.1839 (6)	0.19616 (10)	0.0174 (7)	
O2	0.5793 (5)	0.6847 (7)	0.05563 (12)	0.0308 (9)	
O3	0.6973 (4)	0.4215 (7)	0.10216 (13)	0.0288 (9)	
O4	0.4420 (4)	0.4596 (6)	0.08582 (11)	0.0212 (7)	
N1	−0.0521 (5)	0.0228 (7)	0.05416 (12)	0.0165 (8)	
C1	−0.0322 (6)	−0.1561 (8)	0.03624 (14)	0.0190 (10)	
H1A	−0.1099	−0.2379	0.0248	0.023*	
C2	0.0992 (6)	−0.2181 (8)	0.03460 (14)	0.0200 (10)	
H2A	0.1112	−0.3418	0.0226	0.024*	
C3	0.2145 (6)	−0.0946 (8)	0.05103 (15)	0.0205 (10)	
H3A	0.3045	−0.1329	0.0497	0.025*	
C4	0.1943 (6)	0.0864 (9)	0.06946 (15)	0.0201 (10)	
H4A	0.2718	0.1689	0.0807	0.024*	
C5	0.0596 (5)	0.1481 (8)	0.07152 (14)	0.0159 (9)	
C6	0.0314 (5)	0.3379 (8)	0.09094 (14)	0.0168 (9)	
H6A	−0.0618	0.3793	0.0875	0.020*	
C7	0.1350 (5)	0.4535 (8)	0.11338 (14)	0.0164 (9)	
H7A	0.2272	0.4087	0.1163	0.020*	
C8	0.1164 (5)	0.6439 (8)	0.13376 (14)	0.0160 (9)	
C9	0.2375 (5)	0.7467 (8)	0.15528 (14)	0.0173 (9)	
H9A	0.3262	0.6938	0.1559	0.021*	
C10	0.2276 (5)	0.9264 (8)	0.17581 (14)	0.0175 (9)	
H10A	0.3092	0.9922	0.1901	0.021*	
C11	0.0950 (5)	1.0080 (7)	0.17496 (13)	0.0142 (8)	
C12	−0.0272 (5)	0.9066 (8)	0.15352 (14)	0.0151 (9)	
H12A	−0.1159	0.9596	0.1528	0.018*	
C13	−0.0152 (5)	0.7263 (8)	0.13326 (14)	0.0161 (9)	
H13A	−0.0967	0.6596	0.1191	0.019*	
C14	−0.1979 (5)	0.0792 (9)	0.05387 (16)	0.0207 (10)	
H14A	−0.2612	−0.0282	0.0415	0.031*	
H14B	−0.2233	0.2044	0.0393	0.031*	
H14C	−0.2042	0.0981	0.0806	0.031*	
C15	−0.0362 (5)	1.2805 (8)	0.19457 (14)	0.0174 (9)	
H15A	−0.1005	1.1843	0.2019	0.021*	
H15B	−0.0792	1.3308	0.1681	0.021*	
C16	−0.0034 (6)	1.4575 (8)	0.22382 (15)	0.0211 (10)	
H16A	−0.0891	1.5296	0.2235	0.032*	
H16B	0.0619	1.5497	0.2165	0.032*	
H16C	0.0377	1.4051	0.2499	0.032*	
C17	0.5949 (5)	0.7380 (8)	0.13010 (14)	0.0165 (9)	
C18	0.5583 (5)	0.9431 (8)	0.12275 (15)	0.0194 (9)	
H18A	0.5304	0.9911	0.0969	0.023*	
C19	0.5634 (5)	1.0763 (8)	0.15409 (16)	0.0206 (10)	
H19A	0.5389	1.2137	0.1495	0.025*	
C20	0.6059 (5)	1.0000 (8)	0.19242 (15)	0.0187 (9)	
C21	0.6451 (5)	0.7944 (9)	0.20045 (15)	0.0203 (10)	
H21A	0.6745	0.7465	0.2263	0.024*	
C22	0.6388 (5)	0.6640 (8)	0.16850 (15)	0.0194 (9)	
H22A	0.6641	0.5268	0.1729	0.023*	
O1W	0.2862 (4)	0.4509 (7)	0.00213 (12)	0.0272 (9)	
H2W1	0.3391	0.4482	0.0256	0.06 (3)*	
H1W1	0.3314	0.3960	−0.0129	0.04 (2)*	

### Atomic displacement parameters (Å^2^)

**Table:**

	*U*^11^	*U*^22^	*U*^33^	*U*^12^	*U*^13^	*U*^23^
Br1	0.0304 (3)	0.0266 (3)	0.0250 (3)	−0.0038 (2)	0.0113 (2)	−0.0113 (2)
S1	0.0172 (5)	0.0182 (6)	0.0201 (5)	−0.0051 (4)	0.0070 (4)	−0.0055 (4)
O1	0.0171 (16)	0.0143 (16)	0.0206 (16)	−0.0004 (13)	0.0045 (13)	−0.0053 (13)
O2	0.043 (2)	0.029 (2)	0.0243 (19)	−0.011 (2)	0.0162 (18)	−0.0062 (17)
O3	0.0190 (18)	0.028 (2)	0.038 (2)	0.0020 (16)	0.0049 (16)	−0.0159 (18)
O4	0.0161 (16)	0.0216 (19)	0.0256 (18)	−0.0063 (14)	0.0049 (14)	−0.0051 (15)
N1	0.020 (2)	0.014 (2)	0.0161 (18)	−0.0013 (16)	0.0057 (15)	−0.0011 (15)
C1	0.028 (3)	0.015 (2)	0.015 (2)	−0.0033 (19)	0.0065 (18)	−0.0008 (17)
C2	0.030 (3)	0.014 (2)	0.016 (2)	0.0012 (19)	0.0053 (19)	−0.0025 (17)
C3	0.024 (2)	0.019 (2)	0.019 (2)	0.005 (2)	0.0057 (18)	−0.0003 (19)
C4	0.021 (2)	0.021 (3)	0.018 (2)	−0.0006 (19)	0.0046 (18)	−0.0046 (18)
C5	0.020 (2)	0.013 (2)	0.0142 (19)	−0.0031 (17)	0.0036 (16)	−0.0017 (16)
C6	0.018 (2)	0.015 (2)	0.018 (2)	0.0004 (18)	0.0046 (17)	−0.0012 (17)
C7	0.018 (2)	0.016 (2)	0.016 (2)	0.0005 (18)	0.0070 (17)	−0.0011 (17)
C8	0.018 (2)	0.016 (2)	0.0148 (19)	−0.0022 (17)	0.0051 (16)	−0.0016 (17)
C9	0.018 (2)	0.016 (2)	0.018 (2)	−0.0004 (17)	0.0060 (17)	−0.0034 (17)
C10	0.016 (2)	0.019 (2)	0.017 (2)	−0.0034 (18)	0.0034 (16)	−0.0048 (18)
C11	0.017 (2)	0.013 (2)	0.0131 (19)	−0.0020 (16)	0.0043 (16)	−0.0019 (16)
C12	0.014 (2)	0.016 (2)	0.0152 (19)	0.0006 (17)	0.0043 (16)	−0.0012 (17)
C13	0.017 (2)	0.016 (2)	0.015 (2)	−0.0011 (17)	0.0040 (16)	−0.0018 (17)
C14	0.017 (2)	0.018 (2)	0.027 (2)	−0.0029 (18)	0.0048 (18)	−0.0055 (19)
C15	0.022 (2)	0.012 (2)	0.018 (2)	0.0016 (18)	0.0047 (17)	−0.0020 (17)
C16	0.025 (2)	0.017 (2)	0.021 (2)	0.006 (2)	0.0044 (19)	−0.0040 (18)
C17	0.014 (2)	0.017 (2)	0.019 (2)	−0.0039 (17)	0.0065 (17)	−0.0058 (17)
C18	0.018 (2)	0.020 (2)	0.020 (2)	−0.0010 (19)	0.0049 (18)	−0.0013 (19)
C19	0.018 (2)	0.016 (2)	0.026 (2)	−0.0003 (18)	0.0045 (19)	−0.0038 (19)
C20	0.017 (2)	0.020 (2)	0.020 (2)	−0.0020 (18)	0.0061 (17)	−0.0057 (18)
C21	0.020 (2)	0.021 (3)	0.019 (2)	−0.001 (2)	0.0047 (18)	−0.0016 (19)
C22	0.019 (2)	0.017 (2)	0.023 (2)	−0.0015 (18)	0.0068 (18)	−0.0037 (19)
O1W	0.0217 (18)	0.036 (2)	0.0234 (19)	−0.0020 (17)	0.0056 (15)	−0.0065 (17)

### Geometric parameters (Å, °)

**Table:**

Br1—C20	1.898 (5)	C10—C11	1.397 (7)
S1—O3	1.451 (4)	C10—H10A	0.93
S1—O2	1.452 (4)	C11—C12	1.402 (6)
S1—O4	1.462 (4)	C12—C13	1.393 (7)
S1—C17	1.790 (5)	C12—H12A	0.93
O1—C11	1.365 (6)	C13—H13A	0.93
O1—C15	1.427 (6)	C14—H14A	0.96
N1—C1	1.361 (6)	C14—H14B	0.96
N1—C5	1.374 (6)	C14—H14C	0.96
N1—C14	1.473 (7)	C15—C16	1.520 (7)
C1—C2	1.366 (8)	C15—H15A	0.97
C1—H1A	0.93	C15—H15B	0.97
C2—C3	1.385 (8)	C16—H16A	0.96
C2—H2A	0.93	C16—H16B	0.96
C3—C4	1.383 (7)	C16—H16C	0.96
C3—H3A	0.93	C17—C22	1.387 (7)
C4—C5	1.401 (7)	C17—C18	1.390 (8)
C4—H4A	0.93	C18—C19	1.390 (7)
C5—C6	1.471 (7)	C18—H18A	0.93
C6—C7	1.341 (7)	C19—C20	1.389 (7)
C6—H6A	0.93	C19—H19A	0.93
C7—C8	1.466 (7)	C20—C21	1.402 (8)
C7—H7A	0.93	C21—C22	1.393 (7)
C8—C13	1.393 (7)	C21—H21A	0.93
C8—C9	1.400 (7)	C22—H22A	0.93
C9—C10	1.390 (7)	O1W—H2W1	0.85
C9—H9A	0.93	O1W—H1W1	0.85
			
O3—S1—O2	114.3 (3)	C13—C12—C11	119.7 (4)
O3—S1—O4	113.0 (3)	C13—C12—H12A	120.1
O2—S1—O4	111.7 (3)	C11—C12—H12A	120.1
O3—S1—C17	105.8 (2)	C12—C13—C8	121.3 (5)
O2—S1—C17	105.8 (3)	C12—C13—H13A	119.4
O4—S1—C17	105.3 (2)	C8—C13—H13A	119.4
C11—O1—C15	118.2 (4)	N1—C14—H14A	109.5
C1—N1—C5	121.3 (4)	N1—C14—H14B	109.5
C1—N1—C14	117.7 (4)	H14A—C14—H14B	109.5
C5—N1—C14	121.0 (4)	N1—C14—H14C	109.5
N1—C1—C2	121.5 (5)	H14A—C14—H14C	109.5
N1—C1—H1A	119.2	H14B—C14—H14C	109.5
C2—C1—H1A	119.2	O1—C15—C16	106.1 (4)
C1—C2—C3	119.2 (5)	O1—C15—H15A	110.5
C1—C2—H2A	120.4	C16—C15—H15A	110.5
C3—C2—H2A	120.4	O1—C15—H15B	110.5
C4—C3—C2	119.3 (5)	C16—C15—H15B	110.5
C4—C3—H3A	120.4	H15A—C15—H15B	108.7
C2—C3—H3A	120.4	C15—C16—H16A	109.5
C3—C4—C5	121.3 (5)	C15—C16—H16B	109.5
C3—C4—H4A	119.3	H16A—C16—H16B	109.5
C5—C4—H4A	119.3	C15—C16—H16C	109.5
N1—C5—C4	117.4 (4)	H16A—C16—H16C	109.5
N1—C5—C6	118.7 (4)	H16B—C16—H16C	109.5
C4—C5—C6	123.9 (4)	C22—C17—C18	120.9 (5)
C7—C6—C5	122.6 (5)	C22—C17—S1	118.5 (4)
C7—C6—H6A	118.7	C18—C17—S1	120.6 (4)
C5—C6—H6A	118.7	C19—C18—C17	120.0 (5)
C6—C7—C8	126.1 (5)	C19—C18—H18A	120.0
C6—C7—H7A	116.9	C17—C18—H18A	120.0
C8—C7—H7A	116.9	C20—C19—C18	118.6 (5)
C13—C8—C9	118.4 (5)	C20—C19—H19A	120.7
C13—C8—C7	123.5 (4)	C18—C19—H19A	120.7
C9—C8—C7	118.1 (4)	C19—C20—C21	122.2 (5)
C10—C9—C8	121.2 (5)	C19—C20—Br1	119.0 (4)
C10—C9—H9A	119.4	C21—C20—Br1	118.8 (4)
C8—C9—H9A	119.4	C22—C21—C20	118.0 (5)
C9—C10—C11	119.9 (4)	C22—C21—H21A	121.0
C9—C10—H10A	120.0	C20—C21—H21A	121.0
C11—C10—H10A	120.0	C17—C22—C21	120.2 (5)
O1—C11—C10	115.7 (4)	C17—C22—H22A	119.9
O1—C11—C12	124.7 (4)	C21—C22—H22A	119.9
C10—C11—C12	119.5 (4)	H2W1—O1W—H1W1	107.7
			
C5—N1—C1—C2	−0.3 (7)	O1—C11—C12—C13	−179.3 (4)
C14—N1—C1—C2	178.9 (5)	C10—C11—C12—C13	−0.2 (7)
N1—C1—C2—C3	−1.0 (8)	C11—C12—C13—C8	−0.1 (7)
C1—C2—C3—C4	1.4 (8)	C9—C8—C13—C12	0.1 (7)
C2—C3—C4—C5	−0.6 (8)	C7—C8—C13—C12	179.2 (5)
C1—N1—C5—C4	1.1 (7)	C11—O1—C15—C16	174.6 (4)
C14—N1—C5—C4	−178.1 (5)	O3—S1—C17—C22	−39.4 (5)
C1—N1—C5—C6	−179.1 (4)	O2—S1—C17—C22	−161.1 (4)
C14—N1—C5—C6	1.8 (7)	O4—S1—C17—C22	80.5 (4)
C3—C4—C5—N1	−0.6 (7)	O3—S1—C17—C18	143.5 (4)
C3—C4—C5—C6	179.5 (5)	O2—S1—C17—C18	21.8 (5)
N1—C5—C6—C7	170.2 (5)	O4—S1—C17—C18	−96.6 (4)
C4—C5—C6—C7	−10.0 (8)	C22—C17—C18—C19	−1.0 (7)
C5—C6—C7—C8	−179.9 (5)	S1—C17—C18—C19	176.0 (4)
C6—C7—C8—C13	2.2 (8)	C17—C18—C19—C20	0.2 (7)
C6—C7—C8—C9	−178.7 (5)	C18—C19—C20—C21	0.8 (8)
C13—C8—C9—C10	0.1 (7)	C18—C19—C20—Br1	−179.8 (4)
C7—C8—C9—C10	−179.0 (5)	C19—C20—C21—C22	−0.9 (8)
C8—C9—C10—C11	−0.4 (8)	Br1—C20—C21—C22	179.7 (4)
C15—O1—C11—C10	176.5 (4)	C18—C17—C22—C21	0.9 (7)
C15—O1—C11—C12	−4.3 (7)	S1—C17—C22—C21	−176.2 (4)
C9—C10—C11—O1	179.6 (4)	C20—C21—C22—C17	0.1 (7)
C9—C10—C11—C12	0.4 (7)		

### Hydrogen-bond geometry (Å, °)

**Table:**

*D*—H···*A*	*D*—H	H···*A*	*D*···*A*	*D*—H···*A*
O1W—H2W1···O4	0.85	2.09	2.929 (6)	171
O1W—H1W1···O2^i^	0.85	1.99	2.827 (6)	168
C1—H1A···O1W^ii^	0.93	2.23	3.154 (7)	176
C2—H2A···O1W^iii^	0.93	2.43	3.223 (7)	143
C4—H4A···O4	0.93	2.50	3.378 (7)	158
C6—H6A···O3^iv^	0.93	2.56	3.442 (7)	159
C13—H13A···O3^iv^	0.93	2.49	3.387 (7)	161
C14—H14A···O2^v^	0.96	2.57	3.384 (7)	143
C14—H14C···O3^iv^	0.96	2.51	3.129 (7)	122

Symmetry codes:  (i) −*x*+1, −*y*+1, −*z*; (ii) −*x*, −*y*, −*z*; (iii) *x*, *y*−1, *z*; (iv) *x*−1, *y*, *z*; (v) *x*−1, *y*−1, *z*.
